# Gait in Very Preterm School-Aged Children in Dual-Task Paradigms

**DOI:** 10.1371/journal.pone.0144363

**Published:** 2015-12-07

**Authors:** Priska Hagmann-von Arx, Olivia Manicolo, Nadine Perkinson-Gloor, Peter Weber, Alexander Grob, Sakari Lemola

**Affiliations:** 1 Department of Psychology, University of Basel, Basel, Switzerland; 2 Division of Neuropediatrics and Developmental Medicine, University Children’s Hospital Basel, Basel, Switzerland; Scientific Institute Foundation Santa Lucia, ITALY

## Abstract

**Objective:**

The control of gait requires executive and attentional functions. As preterm children show executive and attentional deficits compared to full-term children, performing concurrent tasks that impose additional cognitive load may lead to poorer walking performance in preterm compared to full-term children. Knowledge regarding gait in preterm children after early childhood is scarce. We examined straight walking and if it is more affected in very preterm than in full-term children in dual-task paradigms.

**Study design:**

Twenty preterm children with very low birth-weight (≤ 1500 g), 24 preterm children with birth-weight > 1500 g, and 44 full-term children, born between 2001 and 2006, were investigated. Gait was assessed using an electronic walkway system (GAITRite) while walking without a concurrent task (single-task) and while performing one concurrent (dual-task) or two concurrent (triple-task) tasks. Spatio-temporal gait parameters (gait velocity, cadence, stride length, single support time, double support time), normalized gait parameters (normalized velocity, normalized cadence, normalized stride length) and gait variability parameters (stride velocity variability, stride length variability) were analyzed.

**Results:**

In dual- and triple-task conditions children showed decreased gait velocity, cadence, stride length, as well as increased single support time, double support time and gait variability compared to single-task walking. Further, results showed systematic decreases in stride velocity variability from preterm children with very low birth weight (≤ 1500 g) to preterm children with birth weight > 1500 g to full-term children. There were no significant interactions between walking conditions and prematurity status.

**Conclusions:**

Dual and triple tasking affects gait of preterm and full-term children, confirming previous results that walking requires executive and attentional functions. Birth-weight dependent systematic changes in stride velocity variability indicate poorer walking performance in preterm children who were less mature at birth.

## Introduction

The incidence of very preterm birth (i.e., birth before the 32nd gestational week) has been rising and survival rates of the very preterm have increased due to improved neonatal care [1]. This development is leading to an increased number of very prematurely born children who are entering public school today. However, even generally well-developing very preterm children who are enrolled in elementary school are at increased risk for several long-term sequels [2], including impairments in cognitive processes such as executive and attentional functions [3–5], and motor development [6], [7].

Motor skills affect important aspects of children’s development [8]. Children with poor motor skills may voluntarily withdraw from situations in which they might show their lower motor abilities [9–12] what may put further motor development at risk [13]. In addition, children with poor motor skills may be perceived by peers as being different or awkward, which may lead to peer rejection [14–16]. Withdrawal from or exclusion by the peer group may both lead to decreased self-esteem, which in turn may increase emotional and behavioral problems [17].

However, although motor impairments have been studied in very preterm children [6], [7], there is scarce of knowledge regarding their development of walking—the most important mode of human locomotion [18].

Gait is a remarkably complex task involving neural control systems to produce coordinated limb movements [19]. Learning to walk freely is a milestone in motor development and requires a learning period of approximately 12 to 14.5 months in full-term infants [20]. Thereafter, gait is continuously stabilized until a mature walking pattern is reached at about 7 years [21]. However, there is evidence that gait continues to develop across childhood and adolescence [22–24]. For example, Hausdorff, Zemany, Peng, and Goldberger [22] showed that gait variability, reflecting the automaticity, rhythmicity, and regularity of gait [25], decreased from childhood to adolescence. In a related vein, Belmonti, Cioni, and Berthoz [24] showed that curvilinear walking, i.e. walking along curved trajectories, is not fully developed before children reach 11 years of age.

Studies with preterm infants indicate that the onset of independent walking is delayed [26–28]. During childhood and adolescence, preterm children show an increased risk for gross motor deficits in dynamic and static balance skills, such as heel walking and one-leg standing [5], [29], and report lower physical activity [30–32] and participation in organized sports [30], [32] than full-term peers. These patterns persist into adulthood [33–35].

Generally, it is acknowledged that executive and attentional functions are involved in the control of gait [36]. Executive functions refer to higher cognitive processes that include the control and allocation of attentional resources necessary for adaptive planning of behaviors [37]. Studies using a dual-task paradigm showed that gait is affected when participants are asked to walk and perform a concurrent task [38]. Studies also revealed that dual-task effects on gait variability are particularly profound in elderly individuals [39] and neurological patients [40–42], who show reduced executive and attentional functions [25], [36], [41–43]. Dual tasking also resulted in gait alterations in children, indicating that gait requires executive and attentional functions also during childhood [44–46]. The effects of dual tasking on gait were more profound in children with deficits in executive and attentional functions, such as children with severe post-traumatic brain injury [47].

In sum, research on gait in generally well-developing very preterm children is scarce. Moreover, the role that limitations in executive and attentional functions play in gait in very preterm children is unknown.

The main goal of the present study was therefore to assess gait and concurrent task performance in very preterm and full-term children in single-, dual-, and triple-task conditions. To achieve ecological validity the dual and triple tasks included tasks that are often required in children's everyday lives while walking, such as listening to someone talking. First, we expected to find impaired gait performance in dual- and triple-task conditions compared to a single-task walking condition in preterm and full-term children. Second, we expected to find more strongly compromised gait performance in very preterm compared to full-term children in dual- and triple-task conditions due to their deficits in executive and attentional functions. Particularly, we hypothesized that very preterm children show higher gait variability than full-term children in dual- and triple-task conditions. Finally, we expected to find decreased performance on the concurrent tasks in preterm compared to full-term children, assuming that concurrent walking would distract their attentional resources more strongly [48].

## Materials and Methods

### Participants

Forty-nine very preterm children were recruited from an initial cohort of 260 very preterm children, born between 2001 and 2006 and postnatally treated at the University Children’s Hospital Basel (Switzerland). One hundred and two preterm children were excluded from the initial cohort due to no or incomplete information on the neurobehavioral development before age 2 or severe developmental delay, insufficient German language skills of parents to give informed consent, or residence outside of Switzerland or more than 100 km away from the study center. Additionally, 7 children could not be traced. Of the 151 parents who were contacted by phone, 49 agreed to allow their children to participate. Compared to nonparticipants, participating preterm children had a higher birth weight (1422 g vs. 1251 g, F(1,150) = 5.46, p = .02), higher gestational age (30.2 weeks vs. 29.3 weeks, F(1,150) = 6.67, p = .01), and a shorter hospital stay (46.5 days vs. 56.3 days, F(1,148) = 5.89, p = .02). Age- and sex-matched full-term children were recruited from birth announcements in newspapers and local schools. As parents of some full-term children refused participation, we were unable to recruit controls for two of the 49 preterm children. Therefore, these two preterm children were excluded from the present study. All children were screened for intellectual disability (IQ ≤ 70) using the German version of the Wechsler Intelligence Scale for Children, 4th edition [49] and for developmental coordination disorder using the German version of the Movement Assessment Battery for Children, 2nd edition with a cut-off below the 16th percentile [50]. None of the children were excluded because of their IQ, whereas 3 preterm children and 1 full-term child were excluded because of significant motor impairment.

The final sample for this study consisted of 44 very preterm children (mean age = 9.48 years, 25 boys, < 32 weeks of gestation, mean birth weight = 1423 g) and 44 full-term children (mean age = 9.47 years, 24 boys, > 37 weeks of gestation, mean birth weight = 3353 g). The sample of preterm children included 20 children with very low birth weight (≤ 1500 g; mean birth weight: 1026 g) and 24 children with birth weight > 1500 g (mean birth weight: 1754 g). None of the children suffered from periventricular leukomalacia, while one of the 44 preterm children was diagnosed with mild intraventricular hemorrhage (IVH) grade 1. All children attended primary school in Switzerland. Preterm and full-term children were comparable regarding age, sex, height, weight, and leg length (Table 1).

**Table 1:** Demographic characteristics of preterm and full-term children.

Characteristic	Preterm			Full Term	A vs. D	B vs. C. vs. D	
	(A) Total (N = 44)	(B) ≤1500 g (n = 20)	(C) >1500 g (n = 24)	(D) Control (N = 44)	*P* Value	*P* Value	Group Differences
Age (years)	9.5 (1.3)	9.7 (1.4)	9.3 (1.3)	9.5 (1.3)	.994	.710	
Male gender	25 (57)	9 (45)	16 (67)	24 (55)	.830	.346	
Gestational age (weeks)	30.1 (2.1)	28.7 (2.3)	31.3 (0.7)	39.6 (1.5)	< .001	< .001	D>B>A
Birth weight (g)	1423 (421)	1026 (250)	1754 (168)	3353 (429)	< .001	< .001	D>B>A
Weight (kg)	32.4 (7.8)	30.2 (7.6)	34.0 (7.6)	33.4 (8.7)	.573	.269	
Height (cm)	139.0 (10.1)	137.6 (11.2)	140.1 (9.3)	138.2 (9.9)	.716	.660	
Leg length (cm)^a^	73.9 (6.9)	73.5 (7.3)	74.2 (6.6)	72.6 (7.5)	.392	.655	
IDS selective attention^b^	120.3 (26.0)	120.6 (27.3)	120.1 (25.7)	131.1 (25.3)	.091	.241	
SDQ hyperactivity/inattention^c^	3.5 (2.3)	3.0 (1.6)	3.8 (2.6)	2.5 (2.4)	.093	.160	

Data are mean (SD) or number (%). *P* values are given for analyses of variance or χ^2^ test. IDS, Intelligence and Development Scales; SDQ, Strength and Difficulties Questionnaire.

^a^ Leg length was measured with footwear from greater trochanter to the floor, bisecting the lateral malleolus.

^b^ Scores range from 0 to 225. Higher scores indicate better performance.

^c^ Scores range from 0 to 10. Higher scores indicate more difficulties.

To screen for executive and attentional functions, we measured selective attention using the selective attention subtest of the Intelligence and Development Scales (IDS) [51], [52]. Additionally, we measured hyperactivity-inattention with the German version of the Strengths and Difficulties Questionnaire (SDQ) [53]. Results revealed a tendency towards lower IDS (p = .091) and higher SDQ (p = .093) scores in preterm compared to full-term children, indicating that executive and attentional functions were marginally lower in the preterm children of our study compared to full-term children.

The Ethics Committee of Basel approved the study, and it was performed in accordance with the ethical standards laid down in the Declaration of Helsinki. Parents gave written informed consent for the children to participate and assent was obtained from the children.

### Procedure and Measures

A portable GAITRite electronic walkway system (GAITRite Platinum; CIR Systems, Sparta, New Jersey) was used for gait assessment. This system consists of a 7.01-m-long electronic mat with 23 040 integrated pressure sensors. Electronically inactive sections with a length of 1.25 m were added on each end of the system to minimize the effects of acceleration and deceleration. The reliability and validity of children’s gait assessment using GAITRite is well established [54], [55]. Analyses were performed according to the European guidelines for spatiotemporal gait analysis [56]. The following spatio-temporal gait parameters were derived: gait velocity (cm/s) which is considered a marker of general functional performance [40] and is the most common reported gait parameter; cadence, measured as steps per minute; stride length (cm) which is the distance between the heel points of two consecutive footfalls of the same foot; single support time (s) which is the time elapsed between the last contact of the current footfall and the initial contact of the next footfall of the same foot; double support time (s) which is the time when both feet are on the floor. In order to account for differences in height of the children we additionally normalized gait velocity, cadence, and stride length to dimensionless quantities using the formulas:

$$\text{Normalized velocity}=\;\frac{gait\;velocity}{\sqrt{\left(g\times l\right)}}$$

$$\text{Normalized cadence}=\;\frac{cadence}{\sqrt{\left(g/l\right)}}$$

$$\text{Normalized stride length}=\;\frac{stride\;length}{l}$$

where *g* is the gravitational constant (9.81 m/s^2^) and *l* is leg length [57], [58]. Finally, we measured gait variability, assessed as variability in stride velocity and stride length using the percentage coefficient of variation (standard deviation/mean × 100). Gait variability is sensitive to more subtle physiological changes and is considered to reflect the regularity, rhythmicity, and automaticity of gait [25]. Correlations between gait parameters in single-task walking are shown in Table 2 and comparable to the correlations in dual- and triple-task conditions (data not shown).

**Table 2:** Correlations between gait parameters in single-task walking (*N* = 88).

		Gait velocity	Cadence	Stride length	Single support time	Double support time	Normalizedvelocity	Normalized cadence	Normalized stride length	Stride velocity variability
1	Gait velocity	1								
2	Cadence	.363**	1							
3	Stride length	.733***	.038	1						
4	Single support time	-.312**	-.953***	-.037	1					
5	Double support time	-.666***	-.477***	-.141	.388***	1				
6	Normalized velocity	.932***	.412***	.525***	-.361**	-.784***	1			
7	Normalized cadence	.802***	.423***	.332**	-.346**	-.662***	.709***	1		
8	Normalized stride length	.614***	.215*	.452***	-.209	-.536***	.790***	.132	1	
9	Stride velocity variability	-.349**	-.044	-.394***	.050	.093	-.283**	-.200	-.234*	1
10	Stride length variability	-.388***	-.008	-.466***	.016	.087	-.309**	-.199	-.275*	.675***

* *p* < .05

** *p* < .01

*** *p* < .001

Prior to gait assessment children were asked to complete all concurrent tasks for 10 s while standing. Concurrent tasks were selected according to related dual-task research and were (1) naming animals at self-selected speed and rhythm (animals) [59], [60]; (2) listening to and memorizing digits (digits) [61], [62], where children heard a word list comprising five digits and five objects as distractors presented in randomized order from a computer over loudspeakers, installed at the front left and right corner of the laboratory. Afterward, the children were asked to recall the digits; (3) carrying a tray (45 × 30 cm) loaded with 7 table tennis balls (tray) [45], [63], [64]. The performance on this task was assessed as number of fallen balls while walking; and (4) unfastening and fastening a button [65], [66] having a diameter of 1 cm (button).

To familiarize the children with the walkway system, each child was given one demonstration and one practice trial before the recordings. A walk was approximately 10 m long and comprised 4 to 5 strides. Then, children were instructed to walk at their normal pace (single-task condition) in four trials. For each child gait parameters were averaged over the trials for further data analyses. Afterward, the children were instructed to walk at their normal pace and simultaneously perform a concurrent task (4 dual-task conditions) or 2 concurrent tasks (3 triple-task conditions) with 1 trial each. While triple-task walking, the children were asked to (1) name animals and carry a tray with balls (animals/tray); (2) listen to and memorize digits and carry a tray with balls (digits/tray) and; (3) listen to and memorize digits and unfasten and fasten a button (digits/button).

We defined “single tasks” as performing only one task at a time (i.e. walking without concurrent tasks or performing a concurrent task without walking). “Dual tasks” were defined as performing two tasks at a time (i.e. walking and one concurrent task such as naming animals), while “triple tasks” were defined as performing three tasks at a time (i.e. walking and two concurrent tasks such as naming animals and carrying a tray). In the dual- and triple-task conditions children were not instructed to prioritize any of the tasks. Task order was not randomized across the study. No randomization was conducted to avoid that some of the children had to perform the same concurrent task in an immediate sequence. Consecutive performance of the same concurrent task (e.g. naming animals) could have led to enhanced learning and memorizing effects in these children.

### Statistical Analysis

First, group differences in gait parameters in the single-task condition were assessed for spatio-temporal gait parameters, normalized gait parameters, and gait variability measures using multivariate analysis of variance (MANOVA). Second, effects of dual- and triple-task conditions on gait were examined using repeated-measures MANOVAs with 3 between-subjects factors (prematurity status: preterm ≤ 1500 g, preterm > 1500 g, full-term) and 8 within-subject factors (walking condition: one single-task, four dual-tasks, three triple-tasks) for each gait parameter. Significant effects were followed up with Bonferroni corrected post-hoc pairwise comparisons in which we focused on single-task versus dual- and triple-task comparisons. Additionally, tests for linear trend by polynomial linear contrast analysis were conducted to test for gait alterations across preterm children with birth weight ≤ 1500 g, preterm children with birth weight > 1500 g, and full-term children. Extreme values in gait parameters defined as a *z* score exceeding 3 SDs from the mean were truncated to ± 3 SD.

Third, a MANOVA was performed to assess group differences in concurrent task performance during single-task condition and, finally, repeated-measures MANOVAs were conducted to examine the effects of dual- and triple-task conditions on the concurrent tasks. Significant effects were followed up with Bonferroni corrected post-hoc pairwise comparisons in which we focused on single-task versus dual- and triple-task comparisons. Tests for linear trend by polynomial linear contrast analysis were employed to test for alterations in concurrent task performance across preterm children with birth weight ≤ 1500 g, preterm children with birth weight > 1500 g, and full-term children.

The normality of distributions of the data was assessed using the Kolmogorov-Smirnov test. As parameters of double support time, gait variability and concurrent task performance were not normally distributed, they were log-transformed. The level of significance was set to .05, with p < .10 considered a tendency. The F statistic, p values (two-tailed), and effect sizes (η^2^) are reported.

## Results

For spatio-temporal gait parameters, means and standard deviations in single-, dual-, and triple-task conditions are shown in Table 3. In the single-task condition the MANOVA showed no significant group differences in these gait parameters (Wilks’ multivariate test, F(10,162) = 0.870, p = .563, η^2^ = 0.051). Repeated-measures MANOVAs revealed a significant within-subject effect of walking condition on each spatio-temporal gait parameter (Wilks’ multivariate test, F(7,73) = 22.983 to 103.825, p < .001, η^2^ = 0.691 to 0.909). Pairwise comparisons revealed lower gait velocity, lower cadence, lower stride length as well as higher single support time and higher double support time in dual- and triple-task conditions compared to single-task walking (p < .01) with exception of the dual-task condition button in which children showed comparable single support time compared to single-task walking. There were no significant main effects of prematurity status or Walking Condition × Prematurity Status interactions.

**Table 3:** Means (and standard deviations) of spatio-temporal gait parameters for preterm and full-term children in single-, dual-, and triple-task conditions.

Gait parameters	Preterm		Full Term (N = 44)
	≤1500g (n = 20)	>1500g (n = 24)	
**Gait velocity (cm/s)**			
Single-task walking	126.61 (15.08)	137.05 (16.73)	132.31 (17.46)
Dual-task walking			
Animals	100.13 (21.18)	102.76 (21.76)	96.87 (20.25)
Digits	97.52 (23.00)	101.30 (15.04)	101.86 (20.25)
Tray	92.93 (21.79)	96.36 (19.31)	100.38 (22.77)
Button	77.84 (20.11)	80.89 (18.30)	82.03 (17.06)
Triple-task walking			
Animals/Tray	81.37 (20.13)	85.04 (19.74)	83.43 (19.84)
Digits/Tray	88.89 (21.10)	90.37 (22.28)	96.30 (16.42)
Digits/Button	86.08 (19.38)	89.20 (16.77)	86.80 (20.32)
**Cadence (steps/min)**			
Single-task walking	124.15 (11.12)	122.08 (10.09)	124.22 (11.37)
Dual-task walking			
Animals	107.40 (15.33)	105.51 (19.01)	105.79 (16.70)
Digits	109.60 (14.09)	107.75 (14.30)	112.83 (10.86)
Tray	112.28 (12.71)	111.92 (13.70)	116.64 (14.04)
Button	100.64 (13.41)	96.20 (11.50)	102.38 (12.56)
Triple-task walking			
Animals/Tray	107.97 (14.17)	101.85 (13.51)	110.68 (11.82)
Digits/Tray	105.28 (15.44)	100.47 (14.23)	104.66 (14.59)
Digits/Button	108.48 (14.68)	106.67 (14.21)	109.40 (14.37)
**Stride length (cm)**			
Single-task walking	125.47 (8.62)	131.95 (12.48)	127.91 (12.21)
Dual-task walking			
Animals	114.88 (14.96)	114.91 (17.41)	110.35 (12.67)
Digits	108.11 (11.19)	111.16 (10.42)	108.41 (11.45)
Tray	100.88 (16.52)	101.47 (16.46)	103.10 (18.24)
Button	95.44 (13.37)	96.86 (14.41)	95.74 (13.23)
Triple-task walking			
Animals/Tray	95.58 (13.37)	97.69 (13.97)	95.35 (15.77)
Digits/Tray	97.35 (11.37)	98.01 (13.07)	94.59 (15.82)
Digits/Button	102.77 (13.17)	101.26 (16.12)	104.33 (12.44)
**Single support time (s)**			
Single-task walking	0.39 (0.03)	0.40 (0.03)	0.39 (0.03)
Dual-task walking			
Animals	0.43 (0.04)	0.47 (0.10)	0.45 (0.09)
Digits	0.43 (0.04)	0.44 (0.05)	0.41 (0.03)
Tray	0.45 (0.04)	0.47 (0.05)	0.44 (0.05)
Button	0.40 (0.03)	0.41 (0.04)	0.39 (0.04)
Triple-task walking			
Animals/Tray	0.42 (0.04)	0.45 (0.06)	0.41 (0.04)
Digits/Tray	0.43 (0.05)	0.44 (0.05)	0.42 (0.04)
Digits/Button	0.41 (0.04)	0.42 (0.05)	0.40 (0.04)
**Double support time (s)**			
Single-task walking	0.19 (0.03)	0.18 (0.03)	0.19 (0.03)
Dual-task walking			
Animals	0.25 (0.06)	0.25 (0.07)	0.26 (0.07)
Digits	0.26 (0.07)	0.25 (0.05)	0.25 (0.04)
Tray	0.28 (0.07)	0.27 (0.08)	0.26 (0.07)
Button	0.33 (0.08)	0.33 (0.09)	0.31 (0.07)
Triple-task walking			
Animals/Tray	0.32 (0.09)	0.31 (0.09)	0.31 (0.08)
Digits/Tray	0.31 (0.09)	0.29 (0.08)	0.30 (0.08)
Digits/Button	0.29 (0.07)	0.30 (0.08)	0.27 (0.06)

*Note*. Animals = naming animals; Digits = listening to and memorizing digits; Tray = carrying a tray with table tennis balls; Button = unfastening and fastening a button.

For normalized gait parameters, means and standard deviations in single-, dual-, and triple-task conditions are shown in Table 4. In the single-task condition the MANOVA showed no significant group differences in these gait parameters (Wilks’ multivariate test, F(6,166) = 0.692, p = .656, η^2^ = 0.024). Repeated-measures MANOVAs revealed a significant within-subject effect of walking condition on each normalized gait parameter (Wilks’ multivariate test, F(7,73) = 37.336 to 95.504, p < .001, η^2^ = 0.784 to 0.902). Pairwise comparisons revealed lower normalized velocity, lower normalized cadence, and lower normalized stride length in dual- and triple-task conditions compared to single-task walking (p < .001). There were no significant main effects of prematurity status or Walking Condition × Prematurity Status interactions.

**Table 4:** Means (and standard deviations) of normalized gait parameters for preterm and full-term children in single-, dual-, and triple-task conditions.

Gait parameters	Preterm		Full Term (N = 44)
	≤1500g (n = 20)	>1500g (n = 24)	
**Normalized velocity**			
Single-task walking	0.47 (0.06)	0.51 (0.07)	0.50 (0.07)
Dual-task walking			
Animals	0.37 (0.08)	0.38 (0.08)	0.36 (0.08)
Digits	0.36 (0.09)	0.38 (0.06)	0.38 (0.06)
Tray	0.35 (0.08)	0.36 (0.07)	0.38 (0.08)
Button	0.29 (0.07)	0.30 (0.06)	0.31 (0.06)
Triple-task walking			
Animals/Tray	0.30 (0.07)	0.31 (0.07)	0.31 (0.08)
Digits/Tray	0.33 (0.08)	0.33 (0.08)	0.36 (0.06)
Digits/Button	0.32 (0.07)	0.33 (0.06)	0.32 (0.08)
**Normalized cadence**			
Single-task walking	0.55 (0.05)	0.57 (0.04)	0.56 (0.05)
Dual-task walking			
Animals	0.48 (0.08)	0.49 (0.08)	0.48 (0.07)
Digits	0.49 (0.08)	0.50 (0.05)	0.51 (0.04)
Tray	0.50 (0.06)	0.52 (0.06)	0.53 (0.06)
Button	0.44 (0.06)	0.46 (0.06)	0.46 (0.06)
Triple-task walking			
Animals/Tray	0.46 (0.07)	0.48 (0.07)	0.47 (0.07)
Digits/Tray	0.48 (0.07)	0.50 (0.06)	0.49 (0.06)
Digits/Button	0.47 (0.07)	0.49 (0.07)	0.50 (0.05)
**Normalized stride length**			
Single-task walking	1.72 (0.16)	1.79 (0.18)	1.77 (0.17)
Dual-task walking			
Animals	1.57 (0.22)	1.55 (0.20)	1.53 (0.20)
Digits	1.48 (0.16)	1.50 (0.13)	1.50 (0.16)
Tray	1.37 (0.18)	1.37 (0.19)	1.42 (0.20)
Button	1.30 (0.21)	1.31 (0.16)	1.33 (0.16)
Triple-task walking			
Animals/Tray	1.31 (0.17)	1.31 (0.16)	1.32 (0.21)
Digits/Tray	1.33 (0.13)	1.32 (0.16)	1.30 (0.20)
Digits/Button	1.40 (0.15)	1.36 (0.18)	1.44 (0.16)

*Note*. Animals = naming animals; Digits = listening to and memorizing digits; Tray = carrying a tray with table tennis balls; Button = unfastening and fastening a button.

Table 5 shows means and standard deviations of gait variability measures in single-, dual-, and triple-task conditions. In the single-task condition the MANOVA showed no significant group differences in the gait variability parameters (Wilks’ multivariate test, F(4,168) = 0.599, p = .664, η^2^ = 0.014). Statistical results from the repeated-measures MANOVAs for each gait parameter are presented in Table 6. For gait variability, results revealed a significant within-subject effect of walking condition on stride velocity variability (Wilks’ multivariate test, F(7,72) = 30.200, p < .001, η^2^ = 0.746) and stride length variability (Wilks’ multivariate test, F(7,72) = 19.003, p < .001, η^2^ = 0.649). Pairwise comparisons revealed higher stride velocity variability and higher stride length variability in dual- and triple-task conditions compared to single-task walking (p < .05) with exception of the dual-task condition digits in which children showed comparable stride length variability compared to single-task walking.

**Table 5:** Means (and standard deviations) of gait variability parameters for preterm and full-term children in single-, dual-, and triple-task conditions.

Gait parameters	Preterm		Full Term (N = 44)
	≤1500g (n = 20)	>1500g (n = 24)	
**Velocity variability**			
Single-task walking	2.81 (0.89)	2.88 (1.11)	2.59 (0.82)
Dual-task walking			
Animals	6.63 (4.36)	8.43 (7.41)	8.15 (7.31)
Digits	4.58 (2.04)	4.37 (2.35)	3.63 (1.55)
Tray	6.39 (3.48)	5.60 (3.40)	6.06 (3.09)
Button	6.78 (5.01)	6.58 (4.70)	5.34 (2.46)
Triple-task walking			
Animals/Tray	5.82 (2.47)	6.46 (4.37)	6.84 (4.71)
Digits/Tray	6.85 (4.24)	5.44 (4.10)	4.91 (2.96)
Digits/Button	5.71 (3.45)	5.33 (4.01)	3.99 (2.28)
**Stride length variability**			
Single-task walking	2.22 (0.70)	2.15 (0.79)	1.99 (0.69)
Dual-task walking			
Animals	4.37 (3.36)	4.81 (4.32)	5.00 (3.91)
Digits	2.58 (1.12)	2.62 (1.53)	2.24 (1.11)
Tray	3.92 (2.59)	4.25 (3.90)	4.67 (2.40)
Button	4.55 (3.42)	4.75 (3.90)	3.64 (1.67)
Triple-task walking			
Animals/Tray	3.70 (1.62)	4.38 (2.72)	4.36 (2.94)
Digits/Tray	3.73 (2.28)	4.10 (2.77)	3.54 (2.07)
Digits/Button	3.66 (2.06)	3.87 (2.94)	2.60 (1.44)

*Note*. Animals = naming animals; Digits = listening to and memorizing digits; Tray = carrying a tray with table tennis balls; Button = unfastening and fastening a button.

**Table 6:** Statistical results from the repeated-measures MANOVAs comparing the single- to the dual- and triple-task conditions for each gait parameter.

Gait parameter	Walking condition			Prematurity status			Walking condition × Prematurity status		
	F	p	η^2^	F	p	η^2^	*F*	p	η^2^
**Spatio-temporal**									
Gait velocity	80.039	< .001	.885	0.973	.383	.024	0.697	.774	.063
Cadence	35.653	< .001	.774	1.133	.327	.028	0.865	.598	.077
Stride length	103.825	< .001	.909	0.359	.700	.009	1.186	.292	.102
Single support time	22.983	< .001	.691	2.265	.111	.055	1.507	.115	.128
Double support time	50.999	< .001	.832	0.599	.552	.015	0.878	.584	.079
**Normalized**									
Velocity	76.647	< .001	.880	0.961	.387	.024	0.697	.774	.063
Cadence	37.936	< .001	.784	1.183	.312	.029	0.635	.832	.057
Stride length	95.504	< .001	.902	0.275	.761	.007	1.277	.228	.109
**Variability**									
Stride velocity	30.200	< .001	.746	2.511	.088	.060	0.890	.570	.080
Stride length	19.003	< 001	.649	0.597	.553	.015	1.058	.400	.093

**Fig 1:**
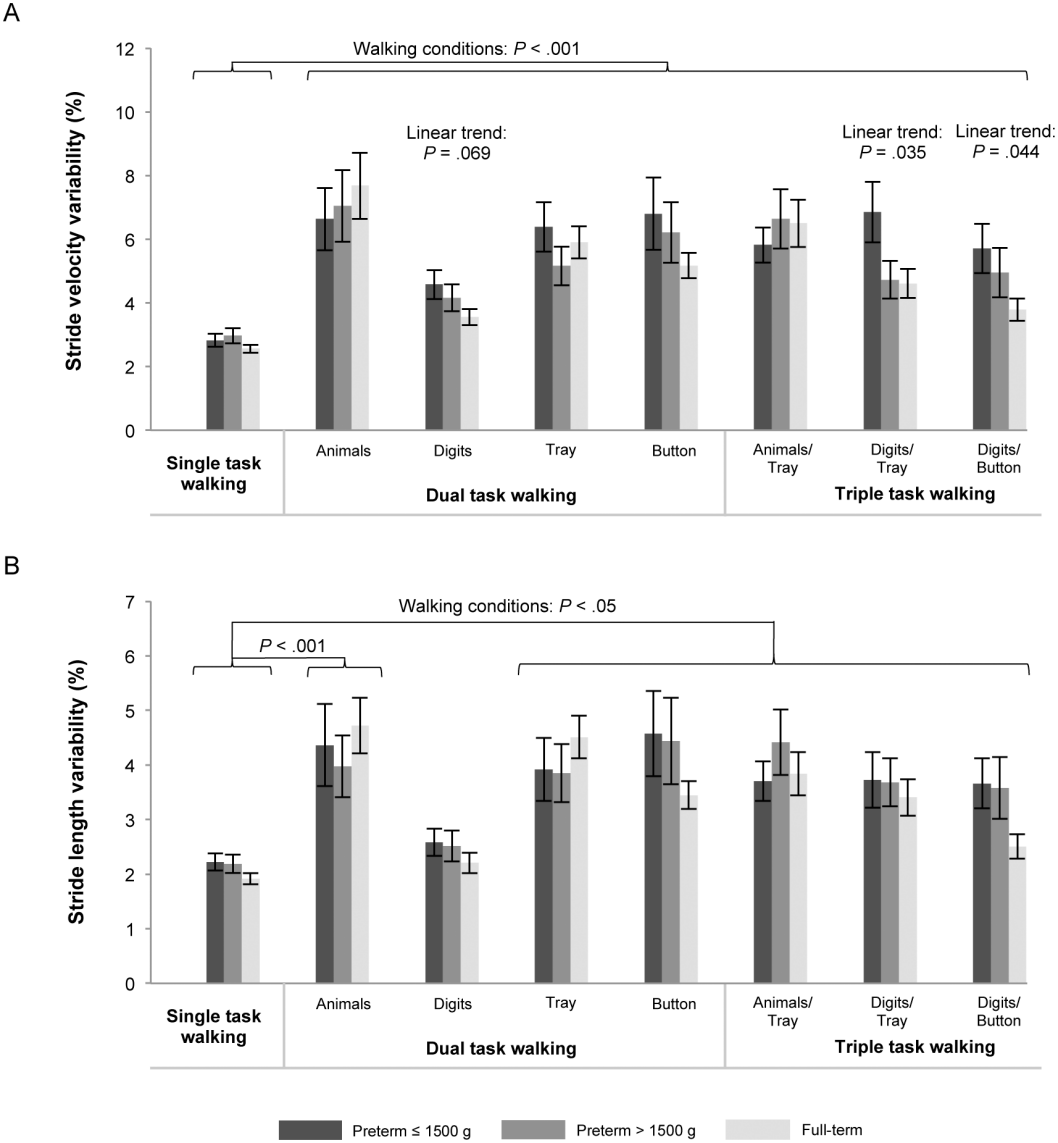
Means and standard errors for gait variability including stride velocity variability (A) and stride length variability (B) for preterm children with birth weight ≤ 1500 g and > 1500 g, and full-term children in single-, dual-, and triple-task conditions. Concurrent tasks were naming animals (animals), listening to and memorizing digits (digits), carrying a tray with table tennis balls (tray), and unfastening and fastening a button (button). *P* values are presented for significant main effects of walking conditions (comparing single task vs. dual and triple tasks) and for linear trends showing increasing gait performance from preterm children with birth weight ≤ 1500 g to preterm children with birth weight > 1500 g to full-term children. For statistical analyses log-transformed parameters of gait variability were used.

For stride velocity variability there was a tendency for a between-subjects effect of prematurity status, F(2,78) = 3.021, p = .088, η^2^ = 0.060, with post-hoc comparisons showing that preterm children with birth weight ≤ 1500 g walked with marginally significantly higher gait variability compared to controls (p = .084). Additionally, polynomial contrasts revealed a linear trend across all walking conditions (p = .028), indicating systematically decreasing gait variability from preterm children with birth weight ≤ 1500 g to preterm children with birth weight > 1500 g to full-term children. The significance of this multivariate linear trend was driven by univariate linear trends in the walking conditions digits (p = .069), digits/tray (p = .035), and digits/button (p = .044), as shown in Fig 1. There were no significant Walking Condition × Prematurity Status interactions.

**Fig 2:**
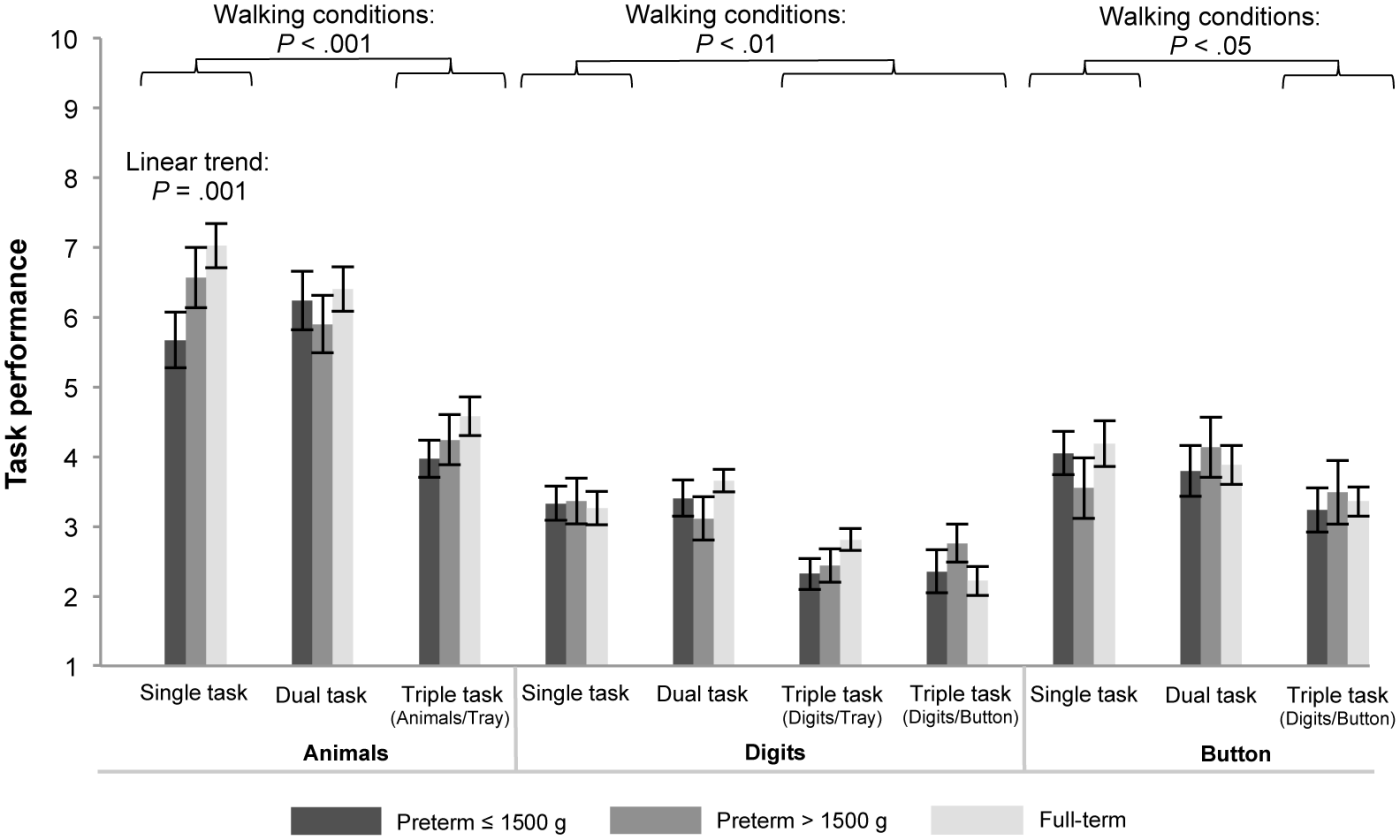
Means and standard errors for task performance for preterm children with birth weight ≤ 1500 g and > 1500 g, and full-term children in single-, dual-, and triple-task conditions. Measures were number of named animals (animals), recalled digits (digits), and how many times a button could be unfastened and fastened (button). Performance in the tray concurrent task was excluded from the figure as both groups showed no variance in this task. *P* values are presented for significant main effects of walking conditions (comparing single task vs. dual- and triple tasks) and for linear trends showing increasing gait performance from preterm children with birth weight ≤ 1500 g to preterm children with birth weight > 1500 g to full-term children. For statistical analyses log-transformed parameters of task performance were used.

Means and standard errors for task performance in single-, dual- and triple-task conditions are shown in Fig 2. As both groups of children showed no variance in the tray concurrent task, it was excluded from further analyses. In single-task conditions the MANOVA revealed a significant effect of prematurity status (Wilks’ multivariate test, F(6,148) = 2.473, p = .026, η^2^ = 0.091). Follow-up univariate tests showed a significant group difference for animals (F(2,76) = 6.277, p = .003, η^2^ = 0.142). Post-hoc pairwise tests showed that preterm children with birth weight ≤ 1500 g named fewer animals than controls (p = .004). Additionally, the test for linear trend was significant, indicating systematically increasing task performance from preterm children with birth weight ≤ 1500 g to preterm children with birth weight > 1500 g to full-term children (p = .001).

Repeated-measures MANOVAs revealed a significant within-subject effect of walking condition on naming animals (Wilks’ multivariate test, F(2,83) = 46.299, p < .001, η^2^ = 0.527). Pairwise comparisons showed that children named fewer animals in the triple-task condition compared to single-task walking (p < .001). There was no main effect of prematurity status nor a significant Walking Condition × Prematurity Status interaction.

Further, there was a significant within-subject effect of walking condition on listening to and memorizing digits (Wilks’ multivariate test, F(3,64) = 16.033, p < .001, η^2^ = 0.429). Pairwise comparisons showed that children recalled fewer digits in the triple-task conditions compared to single-task walking (p < .01). There was no main effect of prematurity status nor a significant Walking Condition × Prematurity Status interaction.

Finally, there was a significant within-subject effect of walking condition on unfastening and fastening a button (Wilks’ multivariate test, F(2,80) = 7.341, p = .001, η^2^ = 0.155). Pairwise comparisons showed that in the triple-task condition children unfastened and fastened a button less often than in single-task walking (p < .05). There was no main effect of prematurity status nor a significant Walking Condition × Prematurity Status interaction.

## Discussion

In everyday life children often do things concomitantly with walking, such as fastening jacket buttons or listening to someone talking, which results in less attention that can be directed to the control of gait. As very preterm children show deficits in executive and attentional functions, the aim of the study was to investigate for the first time gait alterations in dual- and triple-task conditions in very preterm and full-term children during middle childhood.

Our results are consistent with the notion that gait requires executive and attentional functions in children [44–46]: Concurrent information processing in dual- and triple-task conditions interfered with gait, leading to lower gait velocity, lower stride length and higher gait variability compared to single-task walking in both preterm and full-term children.

Regarding prematurity status the results showed systematic decreases in stride velocity variability from preterm children with birth weight ≤ 1500 g to preterm children with birth weight > 1500 g to full-term children, which is in line with research showing that motor impairments occur more frequently in preterm children who were less mature at birth [7], [67]. No significant group differences, however, were revealed in spatio-temporal gait parameters, normalized gait parameters or stride length variability. The result that there were significant group differences in gait variability but not in gait velocity is in line with the notion that gait variability may provide a more discriminant and sensitive measure of gait than other gait variables [25].

Higher gait variability is also present in individuals who exhibit impairments in executive and attentional functions [25], [36], [41–43]. Therefore, deficits in executive and attentional functions in preterm children [3–5] may contribute to the alterations in stride velocity variability in preterm children found in this study. However, the underlying mechanisms for dual task interference in preterm children are not clear. In accordance with the capacity-sharing theory [68] it may be possible that more limited attentional resources in preterm compared to full-term children [69] lead to impaired gait or concurrent task performance as soon as the demands on attention exceed a certain threshold. On the other hand it might be possible that preterm children have more difficulties in switching from one task to the other compared to full-term children [69], which may lead to diminished performance in one or both of the tasks. This notion is derived from the bottleneck theory [70] proposing that two simultaneously performed tasks are cognitively processed sequentially which poses high demands on the capacity to switch between tasks.

In the present study stride velocity variability increased with decreasing maturity of the children at birth, which was most apparent in conditions in which the children had to listen to and memorize digits. This finding appears consistent with results from Huang et al. [46] who found that a concurrent auditory task showed the largest interference effect on gait in five- to seven-year-old children. These authors’ interpretation of their findings was that it is particularly difficult to walk and concurrently perform a task requiring continuous processing of new auditory information. Regarding concurrent task performance, the concurrent performance in naming animals was also related to birth weight such that there was a systematic increase in the number of named animals from preterm children with birth weight ≤ 1500 g to preterm children with birth weight > 1500 g to full-term children, what is in line with previous research [1]. Further, preterm children named fewer animals than full-term children while single tasking. This is in accordance with previous research showing that preterm children scored lower than full-term children in word fluency tests, which are an established measure of executive function [6]. Moreover, it is in line with a recent study showing that the gap in cognitive performance of preterm compared to full-term children systematically increased with increasing cognitive workload of the tasks [71].

However, it remains to be determined why effects of prematurity status were particularly apparent in auditory and verbal fluency conditions. One possible explanation may lie in preterm children’s alterations of interhemispheric integration. Recently, Belmonti, Berthoz, Cioni, Fiori and Guzzetta [72] studied 22 children with cerebral palsy of which 10 children were born premature. In these children locomotor navigation was affected by lesions involving the right frontal lobes, indicating that spatial memory in navigation might depend on right-lateralized networks. Further, there is evidence that cerebral connectivity is altered in auditory language functions in preterm children [73]. Therefore, it is of interest for future studies if such alterations in connectivity in preterm children are also evident in locomotor navigation tasks as they might underlie preterm children's difficulties in walking and concurrently performing an auditory or a verbal fluency task.

From studies with elderly individuals it is known that particularly increased gait variability is associated with a higher risk of falling [34]. However, we are not aware of studies that have examined associations between gait variability and accidents or physical activity in childhood and adolescence. On the other hand, there is evidence that preterm children are less physically active and participate less often in organized sports from childhood through young adulthood [30–35], which may increase their risk for poor cardiovascular outcomes in later life [33]. It is possible that organized sports provide less reinforcing experiences for preterm children due to their more limited walking performance, which may eventually direct them toward less physically active leisure activities. However, the error variance within the assessed groups was high and effect sizes for group differences between preterm and full-term children were small and not consistent across all gait parameters. Therefore, it remains to be shown in future research whether the small differences in gait patterns between preterm and full-term children are meaningful for everyday life and associated with other aspects of children’s development.

Our study has strengths and limitations. We consider it a strength of this study that gait was assessed using an objective electronic gait assessment system with proven reliability and validity [54], [55] for gait assessment when children are wearing their normal clothes and shoes, making it possible to assess children’s gait as it is exhibited in their everyday lives. However, the number of walks per condition was limited and increasing the number of walks might have increased reliability of gait measures. Therefore, replication in other samples of preterm children is important to exclude the possibility that the significant group differences found in the present study were due to the limited number of walks. Further, we analyzed spatio-temporal, normalized, and variability measures of straight walking as the walkway system used in our study did not allow the capture of gait kinematics [74] or the assessment of curvilinear walking [24]. Preterm children participating in our study had higher birth weight and gestational age than nonparticipants, which may lead to underestimation of the effects of dual and triple tasking on gait as cognitive and motor deficits are more profound in less maturely born preterm children [7], [67]. Finally, the cross-sectional design precludes the identification of developmental changes. A longitudinal research approach may be taken to examine whether the gait alterations in very preterm children have to be seen as maturational delay, i.e. that gait matures later in preterm compared to full-term children, or rather as a persistent deviance from the gait pattern of full-term children, i.e. that preterm children do not achieve the maturation of full-term children. In this line, it has been suggested that poorer performance in executive functions of preterm compared to full-term children might reflect a developmental delay rather than a deviance [75].

## Conclusion

Results of this study support the role of executive and attentional functions in the control of gait. Further, the results of our study indicate that preterm children who were less mature at birth walk with higher stride velocity variability. The relevance of these results for everyday motor activity of preterm children as well as whether early developmental intervention training programs focusing on executive function and/or motor behavior [76] could improve gait of preterm children should be the topic of further studies.

## Supporting Information

S1 Dataset Data set used in this study. (SAV)
